# Correlates of prevalent HIV infection among adolescents, young adults, and older adult female sex workers in Ghana: Analysis of data from the Ghana biobehavioral survey

**DOI:** 10.1371/journal.pone.0292686

**Published:** 2023-11-17

**Authors:** Chris Guure, Samuel Dery, Seth Afagbedzi, Ernest Maya, Frances Baaba da-Costa Vroom, Kwasi Torpey

**Affiliations:** 1 Department of Biostatistics, School of Public Health, University of Ghana, Legon, Accra, Ghana; 2 AND Department of Global Health and Population, Harvard T.H. Chan School of Public Health, Boston, MA, United States of America; 3 Department of Population, Family and Reproductive Health, School of Public Health, University of Ghana, Legon, Accra, Ghana; University of Cape Coast Faculty of Social Sciences, GHANA

## Abstract

**Background:**

Human immunodeficiency virus infection remains a high burden among key populations such as female sex workers in the world. We aimed to provide distribution of prevalence and correlates of Human immunodeficiency virus infection among adolescent, young, and older adult FSWs in Ghana.

**Methods:**

This data was obtained from the biobehavioral survey of female sex workers (2020) in Ghana based on a time location sampling approach for the selection of respondents. A sampling frame was developed taking into consideration list of venues, days, and time that sex workers operate across all the regions of Ghana. These lists were derived from a sampling universe which was obtained during a mapping exercise. All sex workers aged 16 years and above and eligible on the day of visit participated. Human immunodeficiency virus testing was done based on First Response and Oraquick. To obtain estimates for sex workers, sampling weights were calculated and applied to the dataset. Inferential analyses using Bayesian regression models were applied with interaction effects.

**Results:**

A total of 5,990 participants completed both the biological and behavioral aspects of the study. The HIV prevalence among female sex workers in Ghana was 4.67% (CI: 4.05%, 5.40%). About 70% of the respondents who tested positive for Human immunodeficiency virus were among the older adults (= >25 years) group. Generally, there was a high prevalence variation across the 16 regions of Ghana, from 0.00% to 8.40%. Respondents’ age was a significant contributor to the prevalence of HIV. Respondents who were forced into having sex had higher odds (38%) of being positive in the combined analysis. Respondents who had comprehensive knowledge of HIV had lower odds (39%) of testing positive.

**Conclusion:**

The findings suggest a low prevalence of HIV among sex workers in 2020 compared to the 2011 and 2015 biobehavioral survey results but higher than the general population. Specifically, older adults have a higher prevalence of HIV. There is generally low level of comprehensive knowledge among sex workers. Interventions geared towards increasing FSW knowledge on risky behavior should be vigorously pursued.

## Background

Female sex work can be classified as direct and indirect. Whereas direct sex work involves either indoor or outdoor exchange of sex for payment where genital contact is normal, indirect sex such as stripping, dancing, phone sex sees less frequent genital contact and a service fee is always charged [1]. Female sex workers (FSW) are a focus group in HIV acquisition, transmission, prevention, and treatment [2–4]. Globally (2019), **female sex workers** have been identified among the population at the highest risk of contracting and spreading the HIV virus [5–7]. Additionally, studies conducted by Shava and others on HIV, confirmed the high incidence and prevalence of the disease among FSW [7–17]. In 2020, key populations (sex workers, gay men, men who have sex with men, transgender people, people who inject drugs, and people in prisons and others) and their sexual partners accounted for 65% of global new HIV infections, of which 8% were sex workers [17, 18]. Sex workers made up 11% of the prevalence of HIV in SSA, whereas FSW account for 12% of HIV infections, their clients and sex partners of all key populations contributed to 19% of the infection [19].The risk of acquiring HIV is higher among FSW than women in the general population. Though key populations constitute a smaller part of the general population, existing analysis suggests that they contribute significantly to the prevalence of HIV infections in sub-Saharan Africa [11, 15, 16].

The HIV and AIDS situation in Ghana is classified as a mixed epidemic having varied and significant prevalence across the different geographical areas [19]. The Ghana AIDS Commission fact sheet 2021, estimates the adult HIV prevalence to be 1.7%. There are significant variations of the HIV prevalence by region. The highest adult HIV prevalence occurred in the Bono region (2.66%) followed by Greater Accra (2.4%) and the least being North East (0.24%), [20]. The prevalence of HIV among female sex workers is consistently higher than the general population. The 2011 and 2015 HIV prevalence for FSW was 11.1% and 70% respectively [21, 22]. Due to the interaction between sex workers (FSW) and the general population, a rise in the prevalence among the general population is highly possible if the situation is not well managed.

There are different predictors of HIV prevalence which depend on country and context. Some of these predictors include inconsistent condom use (people who do not use condoms always) and having multiple sexual partners [12–14]. According to Fauk et al., personal (lack of knowledge of HIV testing and HIV and AIDS service and lack of knowledge of the existence of HIV infection), social (fear of stigma and discrimination), and healthcare provision (lack of trust, fear of disclosure of their HIV status by health professionals and limited availability of medications) barriers associated with HIV testing among male clients contribute to the high prevalence of HIV among FSW [17]. Furthermore, HIV infection is associated with factors such as socio-demographic, socio-economic, and commercial sex work activities and unprotected sex [23]. Some of these factors are mostly due to the inability of FSW to effectively negotiate for safer sex, having sex under the influence of drugs or alcohol, poverty, and high unemployment rates. Female sexual work is primarily to satisfy men’s sexual urges and meet FSW economic needs [23]. However, FSWs are mostly faced with devaluation, disrespect, brutality, social isolation, rejection by others and criminalization which adversely impacts their interactions, work environment and health [24].

A study conducted by Silverman, states that the entry ages of females into sex work varies in every country, whereas some start as early as 10 years, others start at 18 years [25]. According to Su et al., younger age at entry to sex work has a heightened vulnerability to physical and sexual violence victimization which translates to about two to fourfold increase in HIV infection. Though HIV risk reduction among FSW has been a major focus of HIV prevention efforts among implementers worldwide, there is no specific or well-defined public health interventions that addresses HIV infection based on age-groupings [26].

Su et al., showed that age is significantly associated with all measures of HIV risk. According to their findings, older FSWs reported highest rates of lifetime inconsistent condom use, ineffective condom use, sexually transmitted disease (STD) history and lack of HIV testing. This was followed by younger FSWs and then medium-aged FSWs. It was further stated that among the three age groups (≤ 20 years, 21–34 years, and ≥ 35 years), the younger FSWs reported the highest rate of inconsistent condom use in three recent sex acts, followed by older FSWs and then the medium aged FSWs [27]. Studies from China showed that older age was associated with HIV testing [28]; others showed no association between age and HIV risk [29], with others suggesting that older FSWs may be more likely to work in disadvantageous venues, where they may be exposed to HIV risks [30, 31]. Studies in some parts of the world such as India, Spain, and the UK have assessed age-group differences in relation to HIV infection and indicated that younger FSWs (≤20 years) were at higher risk of HIV infection [32, 33].

Although existing studies have revealed different HIV risks among FSWs of different ages, such differences have not been explored in Ghana and so there is no empirical evidence to whether HIV risks differ by age of FSWs in Ghana. The aim of this study is to determine the prevalence and correlates of HIV infection among Ghanaian adolescent (16–19 years), young adults FSW (20–24 years), and older adults, (= >25) female sex workers in Ghana. It is worth mentioning that the population of FSW in Ghana (2020) is 60,049 comprising 55,686 roamers (FSW who are not associated with a specific defined location) and 4,363 seaters (FSW who operate their sex work at specifically defined/fixed and well-known locations), representing 0.76% of the adult Ghanaian female population (15–49 years) [34].

## Methods

### Study design and participants

The survey employed Time Location Sampling (TLS) approach. This method is used when the number of people attending a venue varies over time or day. It is sometimes called venue-day time (VDT) sampling. It is defined according to location (venues where FSW congregate) and time (when the majority of FSW congregate). The sampling frame was developed from the list of VDTs derived from the sampling universe which was obtained during the mapping exercise. Using the data from the mapping and venue verification exercise, the list of all venues or primary sampling units (Venue, Day, and Time—VDT) from the sampling frame (grouped into four-hour intervals for each VDT) as well as a comprehensive sampling frame for each population group at each venue were developed and organized systematically by key geographic units. This mapping strategy was used to develop the sampling frame for the venues in the regions and at the national level. A simple random sample of venues was employed to select from the sampling frame exclusively for unique venues and time.

At each of the VDTs, the field supervisors, with the help of peer educators, counted the number of women present at that venue upon arrival. women who later arrived at the venue after the arrival of the study team were also counted. At the end of the survey, the total number of women who visited the venue were recorded. Almost all women found at the venue were approached using a screening log form, where the number of FSW approached, screened, eligible, willing/interviewed was recorded. All interviews were conducted within a four (4) hour interval with strict adherence.

An FSW is defined as any female aged 16 years (that is, age for consensual sex in Ghana) or older who reports to have exchanged sexual acts (for instance, vaginal, anal and/or oral sex) in the last 6 months with someone other than her established partner for something of value (money and material items) that would otherwise not be extended to them by their sexual partners. This exchange can be either regular or occasional [8]. In the biobehavioral survey, recruitment was only carried out among FSW who self-identified as professional sex worker.

Two main categories of FSW were covered in this study, namely, **seaters** and **roamers**. **Seaters** were defined as FSW who operate at specifically defined/fixed and well-known locations such as homes and brothels including hotels and lodges. These FSW have some form of permanent affiliation with these venues. **Roamers,** on the other hand, are FSW who are not associated with a specific defined location but who usually move from one location to another to seek clients. Their operating venues include bars, hotels, popular eating places and drinking spots, discos, parks, beaches, and street corners. Details of the operationalization and implementation of the study is provided elsewhere, [34].

### Participants recruitment

**Fig 1:**
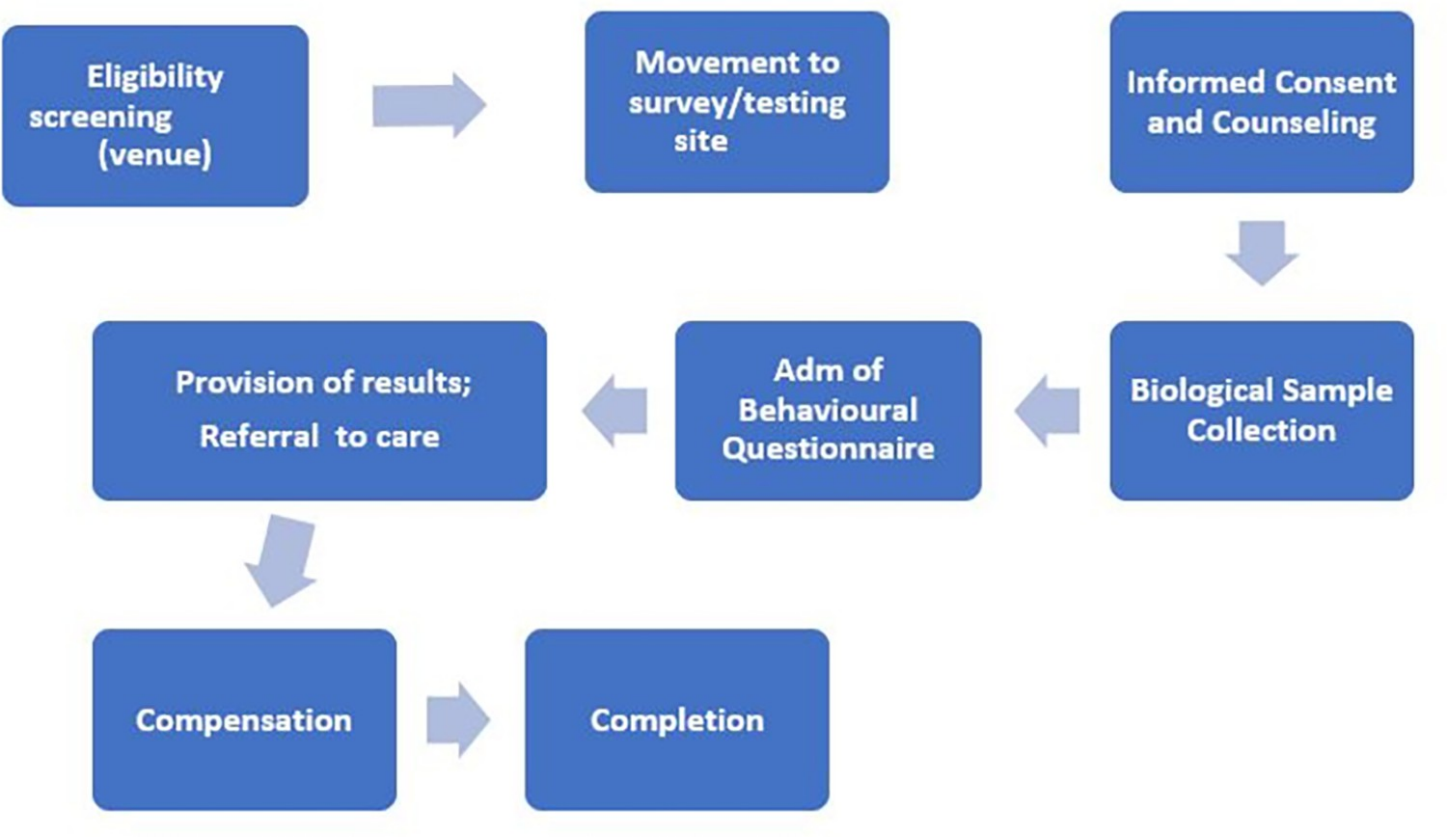
Process of FSW recruitment, interviews, and collection of samples for the survey.

All FSWs who were screened and eligible were taken through the interview process. For each venue, a nearby private room that convenient enough to accommodate both the behavioral and biological teams were acquired for interviews. eligible participants who consented to take part in the study were asked to sign an informed consent form to participate in the interview and collection of blood samples. A complete recruitment process is provided in Fig 1.

### Eligibility criteria and recruitment of FSW

The eligibility criteria were as follows:

1. Born a biological female.
2. Aged 16 years and above.
3. Engaged in sex for money or gift within the last six months.

The research staff explained the study’s rationale to eligible individual participants. For those who consented, a study card bearing a participant ID was issued to the participant and a standardized questionnaire lasting an average of 50 minutes was administered. Overall, an individual participant spent approximately 1 hour and 10 minutes in screening to consent, pre-post counselling, biological and behavioral survey, and collection of on-site results. Study participants who tested positive to HIV were linked to non-governmental organizations (NGO) at their respective regions for onward referral to appropriate treatment facilities. After the linkage, the NGOs submitted the linkage data to the research team members at the University of Ghana. Individual participants were compensated with 50 Ghana cedis irrespective of whether they took part only in the biological, behavioral or both.

### Sample size determination

The sample sizes for both Roamers and Seaters were calculated separately and based on recently tested for HIV among FSW indicator. For roamers, recently tested for HIV was 48% and 61% for seaters from the biobehavioral survey conducted in 2015. The sample size is based on detecting a change of 12 percentage points for both populations with an alpha of 0.03 and 80% power. A design effect of 1.5 and missingness set at 10%. The sample size for seaters was 458 and that for roamers was 515. However, after adjusting for strata (10 regions), the sample sizes were 4,580 for seaters and 5,150 for roamers for the whole country. Based on the 2015 BBS, the estimated total population size of seaters for the 10 regions was 2,319. Therefore, the Finite Population Correction Formula (FPCF) was used with an actual population size of 2,319 in the country. This yielded a sample size target of seaters at 1,540.

### Data collection, tools, techniques and staff

This study was conducted across all the 10 traditional regions of Ghana. A structured questionnaire was developed and reviewed by the study Scientific Advisory Committee. Corrections were effected after the pilot study in three regions (Eastern, Ashanti and Northern) of Ghana. Trained field supervisors approached and recruited the study participants. An online version of the study questionnaire was designed in Research Electronic Data Capture (REDCap) and uploaded onto the REDCap mobile app. This made it possible for the research staff to synchronize data directly to the server. Trained research staff conducted face-to-face interviews with the respondents using the REDCap app. Interviews were conducted from February to March 2020 across all the regions of Ghana. In all, there were 44 supervisors and 166 research staff in the main survey that was carried out across all the 10 traditional (old) regions of Ghana. Research staff were deployed to 392 venues across the country. The research staff positions were advertised **on the notice boards of the University of Ghana School of Public Health** Interviews were conducted and recruitment of staff were based on previous research or work experience with key populations or any relevant public health survey.

### Statistical analysis

#### Sampling weights

To obtain estimates for FSW, sampling weights were calculated and applied to the dataset following the method recommended by University of California San Francisco (UCSF) in TLS guidance. The weight for combining regional data for national estimates was calculated as the inverse of the probability that the FSW was sampled, adjusting for the proportion of venues sampled in the region. Specifically, the national weight was calculated as the inverse of the estimated probability that the FSW was interviewed at the selected venues times and the estimated probability that the venue was selected in the region. The probability of the FSW being selected at the venue was estimated by the percentage of FSW that were interviewed at the venue. This was based on the number of FSW counted at the venue. The probability that the venue was selected was based on the sampling fraction deployed when the random sample of venues in the region was selected. All analyses were weighted.

#### Predicted variables

The predicted variable of interest is HIV status (positive and negative). HIV testing was conducted onsite with whole blood on the First Response HIV 1 and 2 test kit. This rapid diagnostic test enabled simultaneous detection of antibodies specific to HIV (type 1 & 2). Blood samples reactive for HIV were then subjected to the Oraquick HIV-1/2 test kit. Individuals who were HIV reactive on both First Response and Oraquick were classified as HIV positive. A third round of testing was carried out at the Noguchi Memorial Institute for Medical Research for participants who either had their first response or Oraquick to be positive. Those who were positive after the laboratory test, were classified as HIV positive. As part of quality control, all positive cases, and a random sample of 10% of the participants who tested negative were subjected to confirmatory laboratory testing also at the Noguchi Memorial Institute of Medical Research.

#### Segregated target population

The data was grouped into four different populations, that is, adolescent female sex workers aged 16–19 years, young adult female sex workers aged 20–24 years, older adult female sex workers aged = >25 years and the combined data for all age groups.

#### Predictor variables

Variables included and adjusted for in the analysis are, respondent age, educational level, whether the respondent has ever married or not and comprehensive knowledge of HIV. Comprehensive knowledge of HIV was defined as: (i) knowing that both limiting sex partners to one uninfected partner and consistent condom use are HIV prevention methods, (ii) knowing that a healthy-looking person can have HIV, and (iii) rejecting two of the most common misconceptions–that HIV can be transmitted through mosquito bites and by supernatural means. A respondent was classified as having comprehensive knowledge only if she answered correctly to all the above five questions. Further, other variables included, “force sex” (during the last 12 months, did anyone–paying or otherwise–force you to have sex with him when you didn’t want to? “Yes” or “No”), “clients per week” (with how many different people did you have sex in the past one week?), “pay client condom use” (did your most recent paying client use a condom? “used”, “not-used”, “no-response” and “don’t know”), “anal sex” (have you ever had anal sex? “Yes” or “No”), “anal sex riskier” (Is anal sex riskier when it comes to STDs and HIV and AIDS compared to vaginal sex? “Yes” or “No”), “earnings per week” (on average, how much do you earn a week from sex work alone?) and “age started work” (at what age did you start sex work?).

### Data analysis

Descriptive analyses in the form of proportions and mean were carried out separately and in combined form among the specified age groups of female sex workers. Hot Spot Analysis tool was used to calculate the Getis-Ord Gi* statistic (Z-scores) for each FSW venue. This tool works by looking at each feature within the context of neighboring venue HIV+ status. For statistically significant positive z-scores, the larger the z-score is, the more intense the clustering of high HIV+ infections among FSW in the venue. For statistically significant negative z-scores, the smaller the z-score is, the more intense the clustering of low HIV+ infection among FSW in the venue. Inferential analysis between the outcome (HIV status) and socio-economic, demographic, and sexual behavior as well as comprehensive knowledge of HIV transmission variables were examined at the bivariate level to establish associations. Bayesian bivariate and multivariable logistic regression model via the binomial family of distributions were used to examine the association between the outcome and predictors. Four different models were generated taking into consideration the four different age groupings. All variables that showed a significant relationship with the outcome of interest at the bivariate level were used to obtain the adjusted posterior odds ratios (AOR) and credible intervals (CrI). Further to the adjusted analysis, we assessed the presence or otherwise of interaction effects for all variables within each age category and that of the overall analysis. We identified quite several statistically significant interaction effects which were included in the final model. All regression model parameters were assigned flat Normal~ (0, 10,000) priors. We chose flat normal priors due to our lack of prior evidence in Ghana regarding predisposing factors of HIV status among age-specific female commercial sex workers. Two chains were specified with 50,000 simulations, a thinning of 100, and burn-in of 5,000. Convergence diagnostics were carried out using trace plots, autocorrelation plots, histograms as well as the Gelman-Rubin convergence rule. All descriptive analyses were not carried out based on complete case but rather at the variable level with different denominators for each variable just to depict the actual situation as obtained from the field. For the inferential analyses, all participants with missing information were excluded. Statistical significance was determined based on Bayesian credible interval using 95% level. Statistical analyses were completed using Stata version 17.

### Ethical considerations

This study received ethical approval from the University of Ghana Noguchi Memorial Institute of Medical Research Institutional Review Board (CPN 083/18-19), the Ghana Health Service Ethical Review Committee (GHS-ERC 002/05/19) and the Population Council Institutional Review Board (Protocol 891), New York, USA. Study participation was voluntary, and respondents were informed that they were free to withdraw from the study at any time during the survey process. Following careful explanation of the survey, study staff gave eligible respondents the consent form to read or, if necessary, had the consent form read to the respondents by a staff member. All respondents signed that they understood and agreed to all the items contained in the consent form before being enrolled in the survey.

## Results

### Statistical description and distribution of the predicted and predictor variables

**Fig 2:**
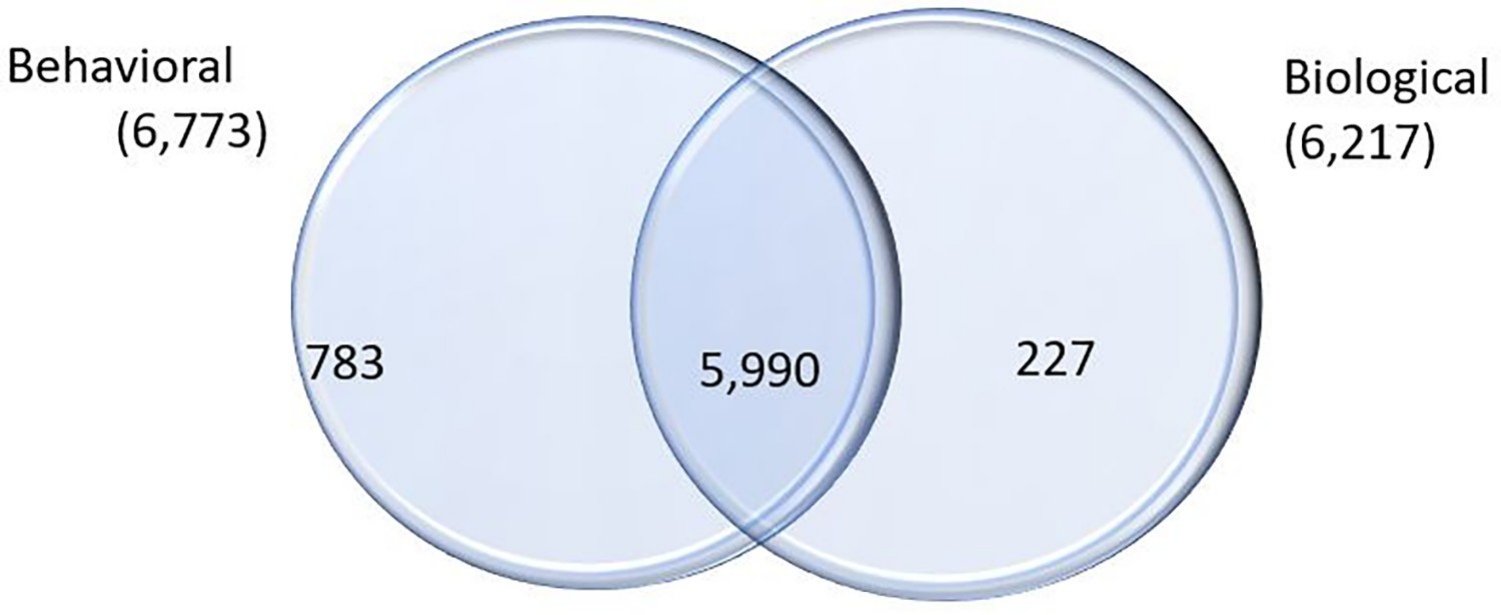
Number of respondents.

There were 7,000 female sex workers who took part in the biobehavioral survey. Out of this number, 6,773 took part in the behavioral survey while 6,217 took part in the biological aspect (gave samples for testing). In all, 5,990 participants completed both the biological and behavioral aspects of the study, while only 783 and 227 took part in only the behavioral and biological aspects, respectively, as shown in Fig 2.

The mean ages of the respondents were 18.17 years for 16–19 group, 22.04 years for 20–24 group, and 30.36 years for = >25 group and 26.43 years for the combined age. Majority 4,776/6,417 (74.11%) of the respondents had completed some secondary education. A total of 2,234/6,445 (34.66%) had comprehensive knowledge with majority 1,356/2,234 (60.71%) of them belonging to = >25-year group. There were about 4,841/6,445 (75.11%) female sex workers who had less than 10 paying clients per week. On average, female sex workers earn GHS469.87 per week, with the = >25-year group earning as high as GHS492.78. A similar percentage of those forced into sex work occurred among the different age groups, although the highest (16.92%) was among young adults (20–24 years). Majority of the respondents 4,910/6,444 (76.19%) started sex work at an age less than 25 years, Table 1. Data was analyzed using information from the behavioral survey.

**Table 1:** Distribution of demographic characteristics of the variables according to age categories (adolescents, young, and older adults) and the combined dataset.

		Age category of respondent		
Variables	(N = 6,445)	n = 665	n = 2,070	n = 3,710
	Combine n(%)	16 to 19 years n(%)	20 to 24 years n(%)	= >25 years n(%)
**Mean age of respondent**	26.43	18.17	22.04	30.36
**Educational level**				
No Education	496(7.70)	29(5.79)	104(20.99)	364(73.22)
Completed Primary	1,172(18.19)	153(13.07)	328(28.02)	691(58.91)
Some Secondary	4,776(74.11)	482(10.10)	1,638(34.29)	2,656(55.61)
**Ever married**				
Yes	2,670(41.54)	188(7.05)	654(24.51)	1,828(68.45)
No	3,723(57.91)	473(12.71)	1,406(37.77)	1,843(49.52)
No response	35(0.55)	1(3.25)	8(21.35)	27(75.40)
**Comprehensive knowledge**				
Yes	2,234(34.66)	202(9.02)	676(30.27)	1,356(60.71)
No	4,211(65.34)	463(10.99)	1,394(33.11)	2,354(55.90)
**Region**				
Greater Accra	732(11.35)	103(15.46)	577(27.88)	1,111(29.96)
Western	609(9.45)	72(10.90)	206(9.96)	453(12.21)
Central	1,791(27.80)	47(7.06)	192(9.27)	370(9.97)
Volta	155(2.41)	30(4.52)	47(2.26)	79(2.12)
Eastern	653(10.13)	142(21.43)	248(12.00)	262(7.06)
Ashanti	1,215(18.86)	110(16.55)	349(16.84)	756(20.39)
Western North	37(0.57)	2(0.30)	13(0.62)	22(0.59)
Ahafo	131(2.04)	7(1.08)	51(2.46)	73(1.97)
Bono	387(6.01)	50(7.52)	113(5.45)	225(6.06)
Bono East	124(1.93)	14(2.17)	51(2.46)	59(1.59)
Oti	166(2.58)	32(4.86)	42(2.05)	91(2.46)
Northern	158(2.45)	12(1.79)	68(3.30)	78(2.10)
Savannah	29(0.45)	1(0.14)	15(0.73)	13(0.35)
North East	30(0.46)	4(0.62)	15(0.75)	10(0.27)
Upper East	161(2.50)	22(3.33)	57(2.75)	82(2.21)
Upper West	65(1.01)	15(2.28)	25(1.19)	25(0.68)
**Force sex**				
Yes	986(15.48)	100(15.12)	347(16.92)	539(14.74)
No	5,382(84.52)	559(84.88)	1,704(83.08)	3,118(85.26)
**Clients per week**				
<10	4,841(75.11)	572(86.15)	1,573(75.99)	2,695(72.65)
11–20	1,067(16.55)	57(8.53)	347(16.76)	663(17.87)
>20	534(8.29)	35(5.31)	149(7.21)	350(9.43)
Non-response	3(0.04)	0(0.00)	1(0.03)	2(0.05)
**Pay client condom use**				
Used	5,761(89.38)	537(81.09)	1,830(88.37)	3,392(91.43)
Not used	625(9.70)	117(17.58)	225(10.89)	283(7.62)
No-response	46(0.71)	6(0.98)	11(0.55)	28(0.75)
Don’t know	14(0.21)	2(0.35)	4(0.19)	7(0.20)
**Anal Sex**				
Yes	656(10.18)	69(10.43)	222(10.70)	365(9.83)
No	5,789(89.82)	595(89.57)	1,849(89.30)	3,345(90.17)
**Anal sex riskier**				
Yes	3,707(57.64)	339(51.07)	1,187(57.39)	2,182(58.97)
No	1,445(22.47)	189(28.48)	459(22.19)	797(21.54)
Don’t know	1,279(19.89)	136(20.45)	422(20.42)	721(19.49)
**Earnings per week**				
Earn week	469.87	347.08	468.27	492.78
**Age started work**				
<25 years	4,910(76.19)	664.27(100.00)	2,070.58(100.00)	2,176(58.64)
25–34 years	1,343(20.84)	-	-	1,343(36.20)
35+ years	191(2.97)	-	-	191(5.16)

The mean age for female sex workers who were HIV positive was 29.09 years and that of negative was 26.33 years. The HIV prevalence among female sex workers was 4.67% with a confidence interval of 4.05% to 5.40%. About 5.70% (186/3,263) of the respondents tested positive for HIV aged = >25 years. The HIV prevalence was 4.22% among the adolescents and 2.93% among the young adults population. More than half 66.03% of the entire FSW population and that of 180/264 (68.14%) of the exclusive HIV positive respondents did not have comprehensive knowledge of HIV. It was observed that FSW without comprehensive knowledge had an HIV prevalence of 4.81% (180/3,750) while those with comprehensive knowledge was 4.37%. Upper East was the region with the highest HIV positive 10/109 (8.40%) female sex workers compared to the other 15 regions. Generally, there was a high HIV prevalence variation across the 16 regions of Ghana, from 0.00% to 8.40%. Out of the 265 respondents who were HIV positive, 155/3,267 (4.74%) said that anal sex was risky regarding HIV infection. The prevalence of HIV among FSW who said anal sex is not risky was 3.40%, while those who engaged in anal sex had a prevalence of 4.38%. Out of the total of 265 HIV positive case, FSW who started sex work less than 25 years were 169 (64.06%) followed by 25–34 years 79(29.73%). The prevalence of HIV was highest (9.33%) among the 35+ year age group, followed by the 25–34 years age group (6.65%), while the lowest was 3.92% among the <24 years group, Table 2. These descriptive analyses were carried out using information from respondents who responded to both the biological and behavioral surveys.

**Table 2:** Distribution of demographic characteristics of the variables according to HIV status.

		HIV Status	
Variables	N = 5,679	n = 5,415	n = 264
	Combine (%)	Negative n(%)	Positive(%)
**Mean Respondent Age**	26.83	26.33	29.09
**Respondent Age**			
<20 years	584(10.28)	559(95.78)	24(4.22)
20–24 years	1,832(32.25)	1,778(97.07)	54(2.93)
= >25 years	3,263(57.46)	3,077(94.30)	186(5.70)
**Educational level**			
No Education	425(7.48)	386(90.71)	39(9.29)
Completed Primary	1,026(18.06)	944(92.00)	82(8.00)
Some Secondary	4,228(74.45)	4,085(96.62)	143(3.38)
**Ever married**			
Yes	2,399(42.55)	2,270(94.62)	129(5.38)
No	3,239(57.45)	3,105(95.86)	134(4.14)
**Comprehensive knowledge**			
Yes	1,929(33.97)	1,845(95.63)	84(4.37)
No	3,750(66.03)	3,570(95.19)	180(4.81)
**Region**			
Greater Accra	1,513(26.64)	1,427(94.35)	85(5.65)
Western	710(12.50)	668(94.16)	41(5.84)
Central	582(10.25)	563(96.73)	19(3.27)
Volta	137(2.41)	130(95.16)	7(4.84)
Eastern	593(10.45)	567(95.55)	26(4.45)
Ashanti	1,043(18.36)	1,002(96.05)	41(3.95)
Western North	36(0.64)	34(93.88)	2(6.12)
Ahafo	111(1.95)	108(97.21)	3(2.79)
Bono	348(6.12)	336(96.55)	12(3.45)
Bono East	120(2.11)	112(93.34)	8(6.66)
Oti	152(2.67)	150(99.13)	1(0.87)
Northern	120(2.11)	116(97.25)	3(2.75)
Savannah	26(0.45)	25(96.14)	1(3.86)
North East	20(0.35)	20(100.00)	0(0.00)
Upper East	119(2.09)	109(91.60)	10(8.40)
Upper West	50(0.88)	47(93.38)	3(6.62)
**Force sex**			
Yes	865(15.41)	817(94.48)	48(5.52)
No	4,748(84.59)	4,536(95.53)	212(4.47)
**Clients per week**			
< = 10	4,276(75.30)	4,081(95.43)	196(4.57)
11–20	922(16.24)	888(96.35)	34(3.65)
>20	478(8.42)	443(92.63)	35(7.37)
No response	3(0.05)	3(100.00)	0(0.00)
**Pay client condom use**			
Used	5,061(89.13)	4,824(95.31)	238(4.69)
Not used	567(9.98)	546(96.33)	21(3.67)
No-response	39(0.68)	36(92.45)	3(7.55)
Don’t know	12(0.21)	9(73.67)	3(26.33)
**Anal Sex**			
Yes	579(10.20)	554(95.62)	25(4.38)
No	5,100(89.80)	4,861(95.31)	239(4.69)
**Anal sex riskier**			
Yes	3,267(57.65)	3,112(95.26)	155(4.74)
No	1,282(22.63)	1,239(96.60)	43(3.40)
Don’t know	1,117(19.72)	1,051(94.10)	66(5.90)
**Earnings per week (GHS)**	469.88	466.78	377.54
**Age started work**			
<25 years	4,321(76.10)	4,152(96.08)	169(3.92)
25–34 years	1,182(20.81)	1,103(93.35)	79(6.65)
35+ years	176(3.10)	159(90.67)	16(9.33)

**Fig 3:**
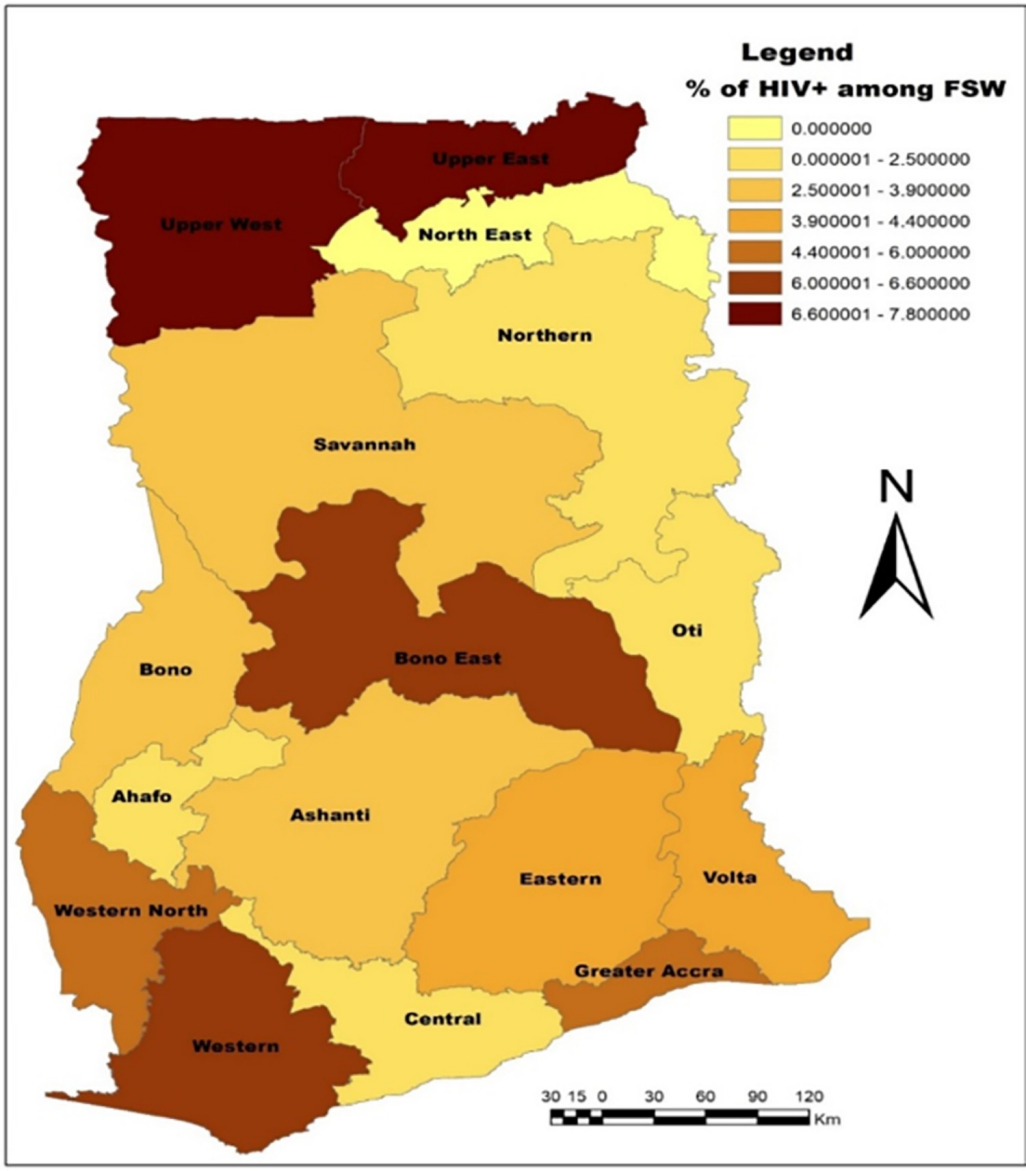
Map of regional HIV prevalence (%) among FSW in Ghana. This map was created using ArcMap 10.7.1 (https://arcgis.com/en/arcmap/10.7/get-started/setup/arcgis.htm).

**Fig 4:**
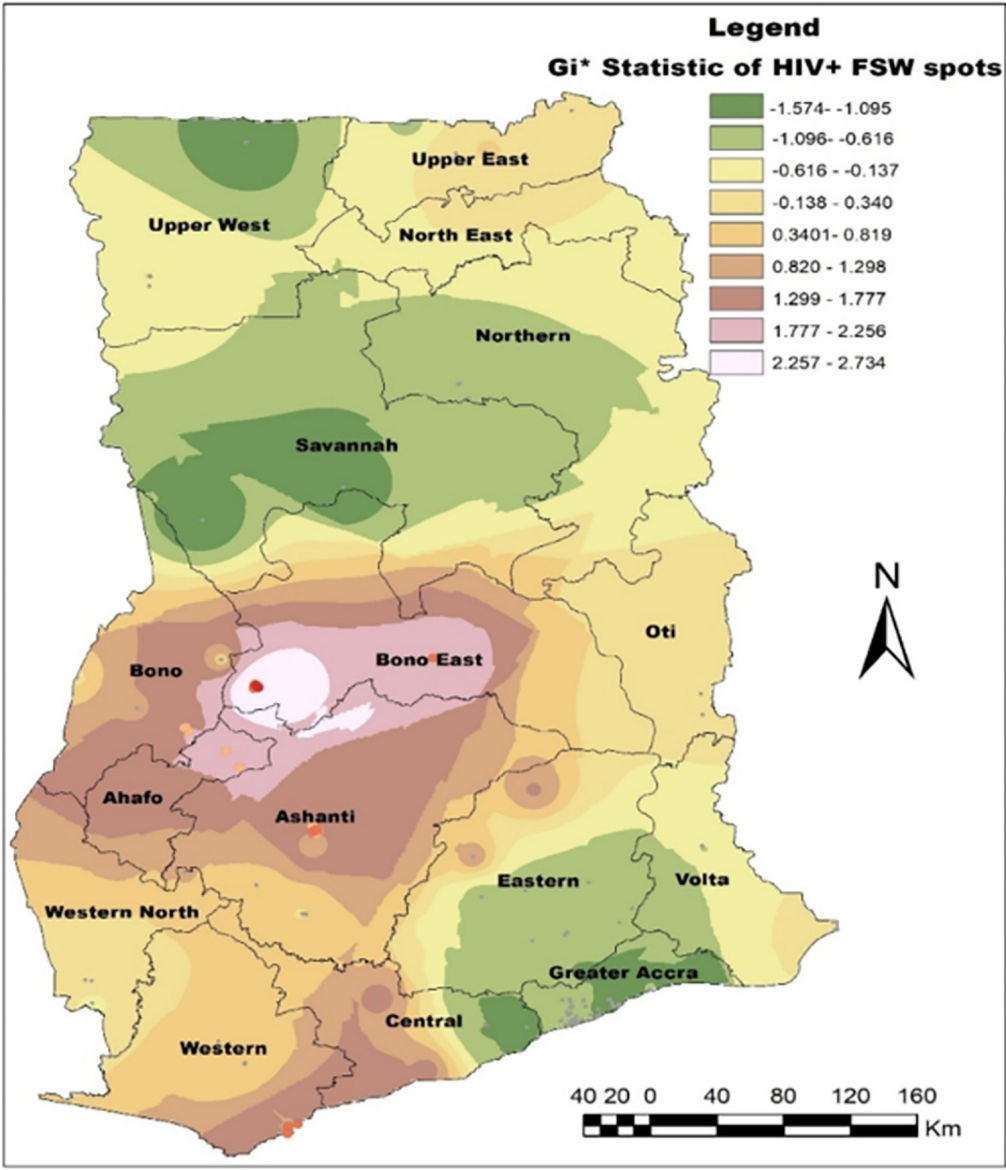
Hotspots of HIV infection among the FSW. This map was created using ArcMap 10.7.1 (https://arcgis.com/en/arcmap/10.7/get-started/setup/arcgis.htm).

We present regional estimates of HIV prevalence (%) among FSW according to the 16 administrative regions in Ghana. The dark brown colours indicate the regions (Upper West and Upper East) with the highest HIV prevalence and light yellowish colours indicate the regions with the least or no HIV prevalence among FSW, Fig 3. Statistically significant hotspots were found in Techiman and Atebubu both in the Bono East region, Kumasi Metropolis in Ashanti, Sekondi Takoradi Metropolis in Western and Sunyani Municipal in the Bono region. Venues with low HIV+ among FSW occurred in Greater Accra, Savannah, and parts of Upper west and Northern regions, Fig 4.

### Adjusted posterior medians (odds ratios) and their highest posterior density credible intervals for adolescents, young adults, older adults, and the combined dataset

With the posterior median estimates for each of the study groups (adolescents, young adults, older adults, and combined), several variables could not be accounted for due to limited responses within the group estimates. Except older adults, all other groups under respondent’s education had lower odds of HIV prevalence, that is, 77% (POR; 0.23, CrI: 0.15–0.31) for adolescents, 81% (POR; 0.19, CrI: 0.13–0.27) for young adults compared to no education. There was a 95% probability that the population odds ratio would lie between 1.46 and 2.73 with an estimated higher posterior odds ratio of 2.05 for respondents who have completed some secondary education compared to those who have never been to school for the older adult population. In the combined data set, we observed that the odds of being diagnosed with HIV among respondents who have either completed primary 25% (POR; 0.75, CrI:0.54–0.89) or obtained some levels of secondary education 77% (POR; 0.23, CrI: 0.17–0.31) were statistically significantly lower compared to those who indicated they have never attended school.

Respondents’ age was a significant contributor to the prevalence of HIV among female sex workers in Ghana (combined data). The posterior median (odds ratio) was 82% (POR; 0.18, CrI: 0.11–0.26) lower for respondents’ who were classified as older adults when compared to the adolescents. Further, 58% lower posterior median with POR of 0.42 (CrI: 0.33–0.53) was observed for those classified under younger adults compared to adolescents. Both of which were statistically significant.

Female sex workers classified as adolescents from Western 66% (POR; 0.34, CrI: 0.24–0.46), Eastern 77% (POR; 0.23, CrI: 0.13–0.39), Ashanti 77% (POR; 0.23, CrI: 0.13–0.37), Bono 74% (POR; 0.26, CrI: 0.18–0.35) and Oti 64% (POR; 0.36, CrI: 0.20–0.55) had a significantly lower posterior odds ratio of being HIV positive compared to those from Greater Accra.

Young adults female sex workers from Central 65%, Eastern 64%, Ahafo 39%, Northern 85% and Savannah 62% were all less likely to be tested HIV positive as against their counterparts from the country’s regional capital (Greater Accra).

For the older adults, there was a statistical significant lower posterior median (odds ratio) among female sex workers who reside in Bono East 43% (POR; 0.57, CrI: 0.41–0.74), Western 36% (POR; 0.64, CrI: 0.47–0.80), Western North 53% (POR; 0.47, CrI:0.20–0.90), Savannah 59% (POR;0.41, CrI:0.22–0.75), Upper East 56%, (POR; 0.44, CrI: 0.39–0.48) and Upper West 20% (POR; 0.80, CrI: 0.70–0.91) regions of being diagnosed HIV positive as against their counterparts in Greater Accra, the capital city of Ghana.

Respondents who were forced to have sex have higher odds 38% (POR; 1.38, CrI: 1.02–1.89) of being diagnosed HIV positive compared to respondents who were not for the combined data. Forced sex was less likely to result in higher HIV positive diagnoses among adolescents (POR; 0.70, CrI: 0.56–0.85) and that of older adults (POR; 0.65, CrI: 0.48–0.85).

Older adults who started sex work at the age of 25–34 years had 2.66-fold the odds of being tested HIV positive against those who started <25 years. For those who started 35 years and above, it was protective compared to <25 years.

Comprehensive knowledge of HIV was observed to be a significant factor across all age groups in lowering the posterior median of HIV prevalence. In the adolescent, young adults, older adults and combined data set, there was lower odds of 48%, 20%, 27% and 39% of respondents who had comprehensive knowledge to test HIV positive compared to those without comprehensive knowledge though it was statistically insignificant among the young adults, Table 3.

**Table 3:** Adjusted posterior medians (odds ratios) and their highest posterior density credible intervals stratified by adolescents, young adults, older adults, and the combined dataset.

Variables	Adolescents OR(CrI)	Young Adults OR(CrI)	Older Adults OR(CrI)	Combine OR(CrI)
**Respondent Age**				
<20 years	ref	ref	ref	ref
20–24 years	-	-	-	**0.42(0.33–0.53)**
= >25 years	-	-	-	**0.18(0.11–0.26)**
**Education Level**				
No Education	ref	ref	ref	ref
Completed Primary	0.76(0.36–1.27)	**0.43(0.36–0.51)**	1.11(0.75–1.56)	**0.75(0.54–0.89)**
Some Secondary	**0.23(0.15–0.31)**	**0.19(0.13–0.27)**	**2.05(1.46–2.73)**	**0.23(0.17–0.31)**
**Region**				
Greater Accra	ref	ref	ref	ref
Western	**0.34(0.24–0.46)**	0.86(0.35–1.54)	**0.64(0.47–0.80)**	1.14(0.81–1.53)
Central	-	**0.35(0.23–0.51)**	0.97(0.60–1.43)	0.73(0.54–1.00)
Volta	0.78(0.43–1.26)	0.77(0.27–1.51)	1.11(0.85–1.41)	0.96(0.71–1.21)
Eastern	**0.23(0.13–0.39)**	**0.36(0.13–0.69)**	0.78(0.49–1.10)	0.82(0.52–1.20)
Ashanti	**0.23(0.13–0.37)**	0.91(0.69–1.18)	1.11(0.80–1.55)	0.86(0.56–1.26)
Western North	**6.19(2.66–11.22)**	0.72(0.41–1.12)	**0.47(0.20–0.90)**	1.59(0.84–2.57)
Ahafo	**-**	**0.61(0.40–0.89)**	**2.72(2.16–3.53)**	0.64(0.26–1.20)
Bono	**0.26(0.18–0.35)**	0.61(0.26–1.12)	1.37(0.80–2.30)	**0.67(0.52–0.85)**
Bono East	1.58(0.88–2.34)	1.36(0.77–2.12)	**0.57(0.41–0.74)**	**1.57(1.01–2.43)**
Oti	**0.36(0.20–0.55)**	-	**7.75(3.53–15.09)**	**0.14(0.09–0.20)**
Northern	-	0.15(0.04–0.33)	1.32(0.72–2.22)	**0.40(0.24–0.54)**
Savannah	-	0.38(0.19–0.61)	**0.41(0.22–0.75)**	0.82(0.44–1.45)
North East	-	-	-	-
Upper East	-	**0.47(0.16–0.95)**	**0.44(0.39–0.48)**	1.16(0.82–1.49)
Upper West	-	1.31(0.37–2.77)	**0.80(0.70–0.91)**	**1.21(0.71–1.85)**
**Ever married**				
No	ref	ref	ref	ref
Yes	**1.49(1.26–1.77)**	0.78(0.52–1.11)	0.95(0.79–1.19)	0.97(0.82–1.18)
**Force sex**				
No	ref	ref	ref	ref
Yes	**0.70(0.56–0.85)**	1.54(0.80–2.56)	**0.65(0.48–0.85)**	**1.38(1.02–1.89)**
**People per week**				
< = 10	ref	ref	ref	ref
11–20	**0.66(0.51–0.86)**	1.02(0.50–1.75)	1.09(0.92–1.22)	**1.48(1.26–1.75)**
>20	**0.61(0.41–0.84)**	0.79(0.60–1.02)	**0.56(0.49–0.64)**	0.94(0.73–1.16)
**Pay client condom**				
Used	ref	ref	ref	ref
Not used	-	-	-	0.88(0.75–1.03)
**Anal Sex**				
Yes	ref	ref	ref	ref
No	**0.33(0.17–0.58)**	0.67(0.33–1.19)	1.00(0.80–1.22)	**0.26(0.19–0.32)**
**Anal sex riskier**				
Yes	ref	ref	ref	ref
No	**2.43(1.11–4.17)**	**0.32(0.14–0.62)**	0.98(0.60–1.57)	1.02(0.59–1.53)
**Age started work**				
<25 years	Ref	ref	ref	ref
25–34 years	-	-	2.66(2.25–3.14)	**0.37(0.22–0.57)**
35+ years	-	-	**0.29(0.22–0.37)**	**3.90(2.81–5.17)**
**Comprehensive knowledge**				
Yes	0.52(0.20–0.96)	0.80(0.42–1.26)	**0.73(0.64–0.84)**	0.61(0.36–0.91)
No	-	-	-	-
**Age started work #Anal sex**				
<25 years	ref	ref	ref	ref
25–34#No	-	-	**0.26(0.16–0.36)**	**3.02(1.71–4.65)**
35+ years#No	-	-	1.90(0.92–3.32)	**0.42(0.33–0.50)**
**educational level# Respondent age**				
Com primary#20–24 years	-	-	-	**0.47(0.24–0.74)**
Com primary# = >25years	-	-	-	1.17(0.89–1.57)
Some Sec#20–24 years	-	-	-	0.69(0.31–1.20)
Some sec# = >25 years	-	-	-	**2.03(1.46–2.74)**
**Respondent age# Anal sex**				
20-24years#No	-	-	-	**3.31(2.23–4.76)**
= >25years#No	-	-	-	**4.91(3.17–7.55)**
**Anal sex# Anal sex riskier**				
No#No	0.35(0.25–0.47)	0.98(0.51–1.57)	1.20(0.58–2.09)	0.67(0.40–1.07)
**Comprehensive knowledge# Respondent age**				
Yes#20 to 24	-	-	-	1.52(0.89–2.34)
Yes# = >25	-	-	-	**1.70(1.24–2.25)**
**Comprehensive knowledge# Age started work**				
Yes#25–34	-	-	-	**1.56(1.00–2.27)**
Yes#35 and above	-	-	-	**1.63(1.03–2.27)**

### Adjusted posterior medians (odds ratio) with interaction effects

Female sex workers who responded ‘yes’ to having had anal sex and having started sex work within the age group of 25 to 34 years were 63% (POR; 0.37, CrI: 0.22–0.57) less likely to be tested positive for HIV. Further, the posterior odds among respondents who started sex work at age 35 and above and have ever had anal sex was about 4-fold statistically significantly higher with a credible interval of 2.81 to 5.17 to testing positive to HIV and those who have never had anal sex and started sex work at the same age 59% (POR; 0.41, CrI: 0.26–0.63) less likely when both are compared to FSW who started at age <25 years. Further, those who did not have anal sex and started sex at age <25 years were 74% less likely to be HIV positive as against those who did have anal sex and in the same age category.

In Ghana, female sex workers who have never had anal sex and are adolescents are 74% (POR; 0.26, CrI: 0.19–0.32) less likely of being tested positive for HIV when compared with those who have ever had anal sex.

Education and age category were observed to be very significant predictors or influencers of respondents who tested positive for HIV. Older adults (= >25 years) who have had some secondary education were 91% (POR; 0.09, CrI: 0.05–0.14) less posterior odds of being tested positive for HIV, while that of older adults who have completed primary school were 84% (POR; 0.16, CrI: 0.09–0.24) less the posterior odds. Only 25% of adolescents were less likely to testing HIV positive. This was in comparison to no education among adolescents. Observably, in the no education category, older adults (82%) and younger adults (58%) were more protective compared to adolescents, Table 4.

**Table 4:** Posterior means (odds ratios) and their highest posterior density credible intervals were obtained for the interaction effects for the combined dataset.

Variables			
	**Anal sex**		
**Age started work**	**Yes**	**No**	
<25 years	ref	0.26(0.19–0.32)	
25–34 years	0.37(0.22–0.57)	0.29(0.19–0.42)	
35+ years	3.90(2.81–5.17)	0.41(0.26–0.63)	
	**Anal sex**		
**Respondent Age**	**Yes**	**No**	
<20 years	ref	0.26(0.19–0.32)	
20–24 years	0.42(0.33–0.53)	0.36(0.21–0.61)	
= >25 years	0.18(0.11–0.26)	0.22(0.15–0.35)	
**Anal sex riskier**	**Anal sex**		
	**Yes**	**No**	
Yes	**ref**	0.26(0.19–0.32)	
No	1.02(0.59–1.53)	0.18(0.13–0.24)	
	**Comprehensive knowledge**		
	**Yes**	**No**	
**Age started work**			
<25 years	0.61(0.36–0.91)	ref	
25–34 years	0.36(0.20–0.62)	0.37(0.22–0.57)	
35+ years	3.87(1.93–7.84)	3.90(2.81–5.17)	
**Respondent Age**			
<20 years	0.61(0.36–0.91)	ref	
20–24 years	0.39(0.23–0.69)	0.42(0.33–0.53)	
= >25 years	0.19(0.10–0.30)	0.18(0.11–0.26)	
**Education level**	**Respondent Age**		
	**<20 years**	**20–24 years**	**= >25 years**
No Education	ref	0.42(0.33–0.53)	0.18(0.11–0.26)
Completed Primary	0.75(0.54–0.89)	0.14(0.08–0.25)	0.16(0.09–0.24)
Some Secondary	0.23(0.17–0.31)	0.07(0.04–0.12)	0.09(0.05–0.14)

## Discussion

The World Health Organization has put into different segments, the vulnerabilities of the world population according to high and less vulnerable towards HIV and AIDS infection [8]. One of the segments of the population vulnerable to high HIV infection is key population within which female sex workers are classified [33]. Studies have demonstrated that age is a significant contributor to HIV infections and HIV preventive practices [35, 36]. This study is the first of its kind to provide prevalence and establish HIV determinants among female sex workers according to adolescents (<20 years), young adults (20–24 years), and older adults (= >25 years) in Ghana. The study goes further and establishes the determinants of HIV infection across the spectrum of female sex workers in the country.

The 2020 biobehavioral survey in Ghana provides a general HIV prevalence of 4.67% among female sex workers. However, comparing the prevalence of HIV (2.1%) among the general population of the country for the year 2020, we observe a much higher prevalence among the female sex workers. A systematic review and meta-analysis from low and middle-income countries found a much higher prevalence of HIV infection among FSW (11.80%) [19]. An observation and comparison of the overall prevalence of HIV among FSW in 2011 and 2015 BBS were calculated to be respectively, 11.1% and 7.0% [22]. This illustrates a decreasing trend of HIV over the years. The drop in HIV prevalence among FSW over the years could be the result of HIV treatment or program coverage and services in the major cities of the country. It might also be because FSWs have taken precautionary measures to curb HIV infection such as condom usage. This could also be because of new uninfected entrants into the sex business.

Furthermore, there is a difference in HIV prevalence among adolescents (4.22%), young adults (2.93%), and older adults (5.70%) FSW. The older adult group had the highest HIV prevalence. The reasons could be that older adults get involved in risky sexual acts and other hindering factors of preventive practices of HIV compared to the adolescent female sex workers. In a related study reported by Sarkar et al., in India, the younger age group of female sex workers were more at risk of HIV infection (12.5%) than the older adults (5.4%) [37]. Younger age group (15–24 years of age) has less HIV preventive practice compared to those in the age group of 25 years and above [36]. The differences identified between the current study and existing literature could be attributed to how age is grouped across studies or because of geographical and cultural factors. Some of these cultural practices that differ across countries include religion, spirituality, medical treatment, emphasis on proper social conduct, respect for elderly and family obligations.

Though comprehensive knowledge of HIV among the FSW population is low (34.66%), It is higher than the general population which was 32.5% in 2014 and 22% in 2020 [22, 38, 39]. A number of studies have demonstrated that comprehensive knowledge of HIV and AIDs is generally low in developing countries [40–42]. Another study conducted by Wang et al., to evaluate HIV-related knowledge and behaviors among people living with HIV (PLHIV) in eight high HIV prevalent countries in sub-Saharan Africa, intimates that comprehensive knowledge was lower in Lesotho, Uganda, and Malawi, similar to the current findings [43]. The adjusted model in this study shows that FSW who had comprehensive knowledge were less likely to be HIV positive. It further showed a significant relationship of reducing the possibility of HIV infection among all specific age groups (adolescents, young adults, and older adults). Comprehensive knowledge was the only consistent modifiable variable that was associated with a decrease in HIV infection among all age groups. In terms of distribution of comprehensive knowledge, it was observed that older adults tended to have higher knowledge followed by younger adults and then adolescent ones. Similar to a study conducted in Zimbabwe among PLHIV clients but contradictory to findings from Lesotho, Malawi, Tanzania, Uganda and Zambia [43]. This indicates that the older FSW might have been exposed to more prevention education and interventions over the years and as such more knowledgeable about HIV infection.

Furthermore, it was observed in this study that, FSW with comprehensive HIV knowledge had a lower HIV infection rate as against those who lack comprehensive knowledge. These findings suggest a negative relationship between HIV infection and comprehensive HIV knowledge. It is possible that, having comprehensive knowledge reduces risky sexual behaviors that may increase the risk of acquiring HIV infection. This in effect leads to the reduction in HIV positive prevalence. It also increases the proportion of people testing for HIV as observed by Wang et al., that there is a positive correlation between prior HIV testing and women’s comprehensive knowledge about HIV and AIDS [43]. This is a positive information for program implementers and policy makers to focus on and allocate more resources in educating this high-risk groups to help reduce the HIV burden among FSW and the country’s general population.

We observed in this study a significant interaction effect between comprehensive knowledge and the age FSW started sex work as well as the educational status of the FSW. The analysis showed higher odds of HIV infection among FSW who had comprehensive knowledge of HIV and started sex work after age 35. Similarly, female sex workers who had satisfactory knowledge of HIV and AIDS were more likely to be infected with HIV, [44]. This may be due to complacency on the part of female sex workers who claim to have good knowledge of HIV transmission practices. The other reason could be that elderly female sex workers may be under pressure to work harder to take care of dependents (single parenting) and so may indulge in risky sexual behaviors due to the higher renumeration that may result from unprotected sex with clients. A similar statistical significance but with negative effect size estimate for HIV infection was observed for the interaction effect between those with comprehensive knowledge and started sex work at age 25–34 years and <25 years. In contrast to these findings, female sex workers aged between 20–24 and 25–29 had higher odds of being infected with HIV and AIDS in Central African Republic, [44].

Furthermore, approximately 15% of FSW responded that they have ever been forced into having sex with their clients. Similar percentage was observed across the different age groups except for the younger adults who were 17%. These estimates are very similar to a study conducted in Ethiopia to examine physical violence and rape among female sex workers [45]. Generally, there was higher posterior odds of being infected with HIV among forced sex clients. Interestingly, among adolescents and older adult population, those who were forced were rather less likely to be infected with HIV. This could mean that during forced sex, either the FSW or their clients find it prudent to protect themselves by wearing condoms. There is also the possibility of female sex workers taking post-exposure prophylaxis after forced sex. We observed from a similar study that looked at the high prevalence of forced sex among non-brothel-based, wine shop-centered sex workers in Chennai, India, and it reported 28% of the respondents experiencing forced sex [46]. In a related study that looked at the living reality of forced sex work, perspectives from young migrant women and sex workers in northern Vietnam, it was found that high levels of forced sex and sexual exploitation were experienced by most of the young women interviewed [47].

Approximately half of the FSW indicated that anal sex was riskier in the transmission of HIV. About 10% of the FSW interviewed in this study have ever engaged in anal sex. This is about half the prevalence as reported in Ahdhra Pradesh in India [48]. Adolescents who did not engage in anal sex were less likely to be infected with HIV. An interaction effect between anal sex and the age in which FSW started sex work and the actual age of the participant were significant. Female sex workers who are not engaged in anal sex and started sex work less than 25 years were not likely to be infected. FSW who reported that they had not had anal sex before and started sex work after 35 years were protective of being infected with the HIV. There was also a high percentage of protectiveness observed among the 25–34 years’ group. Some of the reasons that may influence female sex workers to engage in anal sex even if they are not interested, may include the quest for more money, risk of losing clients and forced sex [47]. A study conducted in Tanzania, stipulates several risky practices associated with anal sex, these include forced sex, multiple partners, low use of condoms, low rates of HIV testing among partakers, poor perception of the risks to acquire HIV through anal sex and use of lubricants [49].

### Limitations

Foremost, our study was cross-sectional in nature and so we are unable to establish causal inferences or relationships of the predictors and the predicted with respect to HIV infections. Further, we acknowledge that our findings may be subject to some level of measurement errors or bias reportage because the information obtained from the female sex workers was self-reported. It is also possible that some sex workers were shy and might have provided responses which they believed were socially desirable to the research team. FSW were provided with a compensation of 50 Ghana cedis after the interview which might have had an influence in their responses.

## Conclusion

This study determined the prevalence and correlates of HIV infection among adolescent female workers, 15–19 years, young adults (20–24) years, and older females (= >25) of FSW in Ghana. FSW who had comprehensive knowledge were less likely to be HIV positive. Comprehensive knowledge was the only consistent modifiable variable that decreases HIV infection among all the age groups. Generally, there was higher posterior odds of being infected with HIV for those who were forced into having sex. Adolescents and older adults FSW who did not engage in anal sex were less likely to be infected with HIV. There is therefore the need for program implementers and policy makers to allocate more resources in educating this high-risk groups to help reduce the HIV burden among them and in the country’s general population.

## Supporting information

S1 Checklist STROBE statement—Checklist of items that should be included in reports of observational studies. (DOCX)
